# Close Margins in Oral Cancers: Implication of Close Margin Status in Recurrence and Survival of pT1N0 and pT2N0 Oral Cancers

**DOI:** 10.1155/2014/545372

**Published:** 2014-11-11

**Authors:** Sandhya Gokavarapu, Ravi Chander, Nagendra Parvataneni, Sreenivasa Puthamakula

**Affiliations:** ^1^Basavatarakam Indo American Cancer Hospital and Research Centre, Hyderabad, Andhra Pradesh 500034, India; ^2^Seven Hills Hospital, Andheri West, Mumbai, India

## Abstract

*Introduction.* Among all prognostic factors, “margin status” is the only factor under clinician's control. Current guidelines describe histopathologic margin of >5 mm as “clear margin” and 1–5 mm as “close margin.” Ambiguous description of positive margin in the published data resulted in comparison of microscopically “involved margin” and “close margin” together with “clear margin” in many publications. Authors attempted to compare the outcome of close and clear margins of stage I and stage II squamous cell carcinoma of oral cavity to investigate the efficacy of description of margin status. *Patients and Methods.* Historical cohorts of patients treated between January 2010 and December 2011 at tertiary cancer hospital were investigated and filtered for stage I and stage II primary squamous cell carcinomas of oral cavity. Patients with margin status of tumor at margin or within 1mm from cut margin were excluded and analyzed in multivariate logistic regression model for locoregional recurrences and Cox regression for overall survival. *Results.* A total of 104 patients fulfilled the abovementioned criteria, of whom 36 were “clear margin” and 68 were “close margin” with median period of follow-up of 39 months. There was no significant difference in locoregional recurrence (*P* value: 0.0.810) and survival (*P* value: 0.0.851) among “close margin” and “clear margin” patients.

## 1. Introduction

Globally lip and oral cancers together comprise of 9.7% of all the cancers [1]. Incidence of oral cancer is much higher in developing countries than developed countries. They comprise one-third of all cancers in southeast Asia [2]; the higher incidence is attributed to more popular chewable forms of tobacco in this region [3]. About 90% of oral cavity tumors are squamous cell carcinomas [4].

Surgery is primary treatment modality and best choice in oral cancers owing to anatomical considerations of complex bone and soft tissues in this area [5]; moreover morbidity associated with primary radiotherapy on quality of life and persistent xerostomia is considerable [6].

Prognostic risk factors of oral cancer include tumor staging and grading, marginal status, lymph vascular invasion, perineural spread, and perinodal spread of regional disease, of which marginal status is the only factor to a variable extent under clinician's control [7].

Although surgeon always aims at a resection with clear margin, close margins are inevitable; complexity of oral anatomy explains the fact that positive margins are most frequent in oral cancer resection in comparison to the cancers of upper aerodigestive tract [8–11].

Intraoperative tools such as use of frozen section may benefit in some patients to identify involved margin for further revision but fail to identify close margins [12]. Close margins are possible to be evaluated only after complete sectional evaluation in histopathology; such processing is not possible in short intraoperative period. Currently, there is uniform consensus regarding marginal status. Margins are regarded as clear when histological margin from invasive carcinoma was more than 5 mm, 1–5 mm distance was regarded as close, and less than 1 mm was regarded as involved [13].

Considerable controversy exists on the criteria for positive margins. Looser et al. [14] have made a classification of positive margins on four histological criteria: (1) margin closeness (tumor within 0.5 cm), (2) premalignant change in margin, (3) in situ cancer in the margin, and (4) invasive microscopic cancer in the margin. Loree and Strong [15] considered lesion tissue within 0.5 mm of the surgical margin with the exception of laryngeal lesions, dysplastic epithelium at the margin, carcinoma in situ at the margin, and invasive carcinoma at the margin to be positive margins.

In patients habituated to tobacco and betel chewing, entire mucosa undergoes premalignant change. Pindborg and Sirsat in their evaluation of 89 OSMF (oral submucous fibrosis) patients described coexisting oral cancer and dysplasias [16]; Chaturvedi et al. reported coexisting OSMF in 30% of oral cancer patients in India [17]. Trismus and difficulty in access to tumors in these patients increase the possibility of close resection margins; often these patients undergo extensive resections for low tumor volume in an attempt to gain oncologic safe margin. Considering dysplasia and premalignant change to be a positive margin would result in excessive reporting of positive margins and comparison of these patients with patients of involved margin; moreover, this necessitates adjuvant therapy in most of these patients. Anneroth et al. [18] have discussed the reasons behind the varying results obtained in studies using histomorphologic grading schemes and the potential errors in clinical research associated with oral cancer.

Because of all the abovementioned factors, we investigated patients with close margins free of tumor and analyzed them with clear margin cases for overall outcome.

## 2. Patients and Materials

A total of 563 primary oral cancer patients were treated surgically in this period; after exclusion of patients with nonsquamous cell carcinoma, carcinoma of lip, and recurred, residual, and second primary oral cancers, 457 patients were isolated: 148 patients were lost to follow-up during this period and 309 primary oral squamous cell carcinomas were filtered (the demographics of patients such as age, sex, and stage of disease were comparable in the patients with and without continued follow-up; follow-up was lost due to disconnection in the contact telephone number in most of the cases); among these patients, cases with verrucous and hybrid carcinomas were excluded along with patients with tumor within 1 mm from cut margin; based on pathological staging, stage I and stage II cases (UICC TNM Staging) with close and clear margins were 104 in number. The median period of follow-up was 37 months.

Patients with margin status of less than or equal to 5 mm were regarded as “close” and greater than 5 mm as “clear.” All the patients had undergone neck dissections in the selected sample and pathological staging depended on the same.

Variables were first tested for associations with outcome, that is, “locoregional recurrence” and “death” in univariate analysis using the chi-square test or Fisher's exact test and log-rank test as appropriate. Variables associated with outcome (*P* < 0.10) were further tested in a multivariable Cox regression model and multivariate logistic regression model adjusting for potential risk factors and confounders. The Kaplan-Meier graphs were drawn to indicate the survival probability. The software SPSS version 17.0 for Windows (SPSS, Chicago, IL, USA) was used for statistical analysis. A two-tailed *P* value of less than 0.05 was considered statistically significant.

## 3. Results

Among a total of 104 patients, 71 (68.2%) were male and 33 (31.7%) were female in the sample. The mean age was 49.6 years and range of age 21 years to 79 years; 93 (89.4%) survived to date and eleven patients died.

52 patients received PORT (postoperative radiotherapy); a dose of 56 gray was delivered most often. There was no significant difference in the outcome (locoregional recurrence: *P* value = 0.288 [chi-square test] and death *P* value = 0.26 [log-rank test]) among the patients in close margin group who received PORT to that of patients who did not receive PORT.

Demographic and prognostic factors in either of the groups are illustrated in Table 1.

In the univariate log-rank test, patients graded MDSCC (moderately differentiated squamous cell carcinoma) had higher risk of death (*P* = 0.008) to patients with WDSCC (well differentiated squamous cell carcinoma). The patients with stage II disease had higher risk of death (*P* = 0.017, Table 2).

In the multivariable Cox regression model, after adjustment for tobacco use, margin status and perineural invasion, patients diagnosed with MDSCC and patients with stage II disease had higher risk of death and adjusted hazard ratio (HR): 4.89 (95% CI: 1.19, 20.13) (*P* = 0.028) and 6.20 (95% CI: 1.27, 30.21) (*P* = 0.024), respectively (Table 3).

In the univariate analysis, patients with stage II disease significantly had higher risk of recurrence when compared with stage I disease (*P* = 0.046, Table 4). Patients diagnosed with MDSCC had higher loco recurrence but it was borderline significant risk (*P* = 0.062) (Table 4).

In the multivariate logistic regression model, patients with stage II disease had higher recurrence and adjusted odds ratio (OR): 5.41 (95% CI: 0.99, 29.36) (*P* = 0.050, Table 5); however, it was borderline statistically significant. Patients diagnosed with MDSCC had higher locoregional recurrence, OR: 4.21 (95% CI: 0.89, 19.93) (*P* = 0.070); however, this association was borderline statistically significant. The multivariate logistic regression model built in this instance has best fit according to Hosmer and Lemeshow test (*χ* ^2^ = 7.00; *P* = 0.537, Table 5).

**Figure 1:**
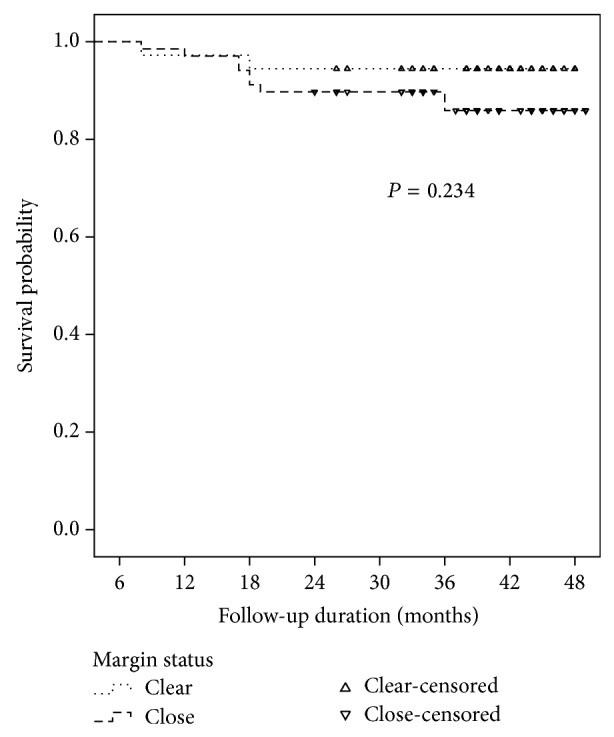
The Kaplan-Meier graph indicating survival.

The Kaplan-Meier graphs for survival did not show any significant difference in either of the groups, Figure 1.

## 4. Discussion

Margin status has been controversial throughout the literature; various variables regarding margin status include (1) necessary distance of the cut margin from the tumor, (2) definition of positive margin, (3) implication of premalignant change in the margin, and (4) management of close margins by PORT.

Adequacy of margin is an important prognostic factor, but what is an adequate margin? Few researchers have recommended up to 2 cm clinical margin, whereas according to few as much as 3 mm was sufficient [19, 20]; currently maximum uniformity is “1 cm three-dimensional margin” [21]. This should be reflected into >5 mm of pathological margin [22].

Shrinkage of the tissues is of concern to obtain optimal pathological margin; the greatest proportion of shrinkage occurs immediately upon resection [23]. Fixing of specimen results in 40 to 50% of shrinkage [22]. Mistry et al. [24] reported that the mean shrinkage in T1/T2 tumors (25.6%) was significantly more than that in T3/T4 (9.2%, *P* < 0.011). Johnson et al. [23] observed that the mean shrinkage of the labiobuccal mucosal margin was 47.3% (*P* < 0.0001). González Ballester et al. [25] evaluated the shrinkage and measurement of marginal status at various steps; they noted that there is discrepancy in the measurement of margin in freshly fixed tissue to that of microscopic examination wherein there was considerable amount of shrinkage and cautioned against adjuvant radiotherapy resulting in increased morbidity. This implies that after thorough consideration of shrinkage of mucosa in the process of processing and fixing 5 mm margin is sufficient. This does not hold for bony margins since they do not shrink. If we consider that maximum 50% of shrinkage is possible [21], then necessary clinical margin for resection should be greater than 1 cm for mucosal and deep margins and 1 cm for bony margins to obtain overall margin greater than 5 mm.

Nason et al. [19] reported equal survival and recurrence rates among the patients with 3 to 4 mm margin with that of 5 mm or more margins in his systematically evaluated Cox proportional hazard model. He stressed the necessity of reassessment on close margins clear of tumor. Barrya et al. [20] evaluated local recurrence in 2 groups, one with pT1,T2 N0, and pT1, pT2 regardless of neck status or radiotherapy; they did not find any statistical difference between both of the groups for margins 3 mm to 4.9 mm and greater than 5 mm cases. Alicandri-Ciufelli et al. [26] defined close margin in oral cancer as less than or equal to 4 mm in their systematic evidence based review of margins in head and neck cancer.

In the current study, there was no significant difference between locoregional disease recurrence and survival among patients with clear margin and close margin. This strengthens the observations of Batsakis [22], Nason et al. [19], and Barrya et al. [20] who pointed out the necessity of reevaluation of defining 5 mm as close margin. Though many of our patients received PORT among “close” margin patients, PORT did not appear to improve survival since 17% (*n* = 6) of patients died in patients of close margin who received PORT (*n* = 35) whereas 9% (*n* = 3) of patients died in patients of close margin who did not receive PORT (*n* = 33).

It is difficult to compare our results with the earlier researchers since the results they have mentioned included patients with close margin along with the group of patients having microscopically involved margin and called them “positive” margin.

Published data on the management of premalignant change in the margin including dysplasia is complicated. Meier et al. [27] in their survey on current clinical practices in head and neck cancers have reported that 76% of International American Head and Neck Society members who participated in the survey considered dysplasia in the margin to be negative. It is clear that there is no uniformity in consensus on what is regarded as a positive margin; the data on the prognosis of close margins or positive margins is variable depending on the criteria the researcher has chosen; we did not consider dysplasia in the margin positive since the prevalence of premalignant condition such as OSMF is high in patients diagnosed with oral cancer [17]; field changes in the entire mucosa are more frequently observed [16]; considering premalignant change and dysplasia in the margin to be positive would result in justifying PORT in these patients in whom morbidity associated with RT is much severe due added effect of radiation induced fibrosis [28] to previously existing submucous fibrosis. Premalignant change in the margin was considered a risk factor in the development of second primaries [29] rather than inadequate clearance of primary tumor. However, carcinoma in situ at margin was regarded as involved margin and such patient was not included in the study.

Published data regarding weather PORT should be given in patients with close margin is complicated; since initial research included these patients in the group of patients with positive margins, they justified PORT to improve survival. But recent publication by Barrya et al. [20] in which author compared recurrences between pT1, T2 N0 and pT1, pT2 regardless of neck status or radiotherapy did not find any statistical difference between patients with margin of 3.0–4.9 and >5.0. Wong et al. [30] suggested that surgical margins within 2 mm should be considered the cut-off for recommendation of PORT. Ch'ng et al. [31] also have concluded that patients with close margins had acceptable local control without PORT in the absence of other risk factors.

52 patients in current study received adjuvant therapy; however, univariate analysis did not show influence of PORT on outcome.

Close margins did not significantly affect the locoregional recurrence or survival; published data was variable regarding the prognosis of close margins. Selection bias is possible wherein majority of the patients in a study may be in lower end of range of 1 mm to 5 mm or higher end of the same range. Recent data on margins distinguishes different survival among patients below and above 3 mm margin [18]. Current study highlights the deficiencies of existing criteria for the definition of close margin.

Current study is a retrospective study; all the prognostic factors may not be evaluated; however, a prospective study in this regard might not be possible for ethical considerations; thus the information obtained from such data might help clinician to understand the implications of close margin regarding recurrences and survival. Moreover, this study included primary pT1N0, pT2N0 tumors only and excluded verrucous or hybrid carcinomas. The cases in the study are uncomplicated with regional disease and large surface areas of T3 and T4 tumors.

## Figures and Tables

**Table 1:** Demographic, prognostic factors of the sample.

Factors	Total sample *N*	Close margin (group 1)	Clear margin (group 2)
Site			
Tongue	72	40	32
Buccal mucosa	28	24	4
Gingiva	4	4	0
Tobacco use			
No	44	28	16
Yes	60	40	20
PORT			
No	52	33	19
Yes	52	35	17
HPE diagnosis			
WDSCC	73	44	29
MDSCC	31	24	7
Lymphovascular spread			
Absent	103	67	36
Present	1	1	0
Perineural invasion			
Absent	100	65	35
Present	4	3	1
T stage			
T1	54	30	24
T2	50	38	12

**Table 2:** Univariate analysis of factors associated with survival.

Factors	Total sample *N*	Death *n* (%)	P value
Site			
Tongue	72	8 (11.1)	0.785
Buccal mucosa	28	3 (10.7)	
Gingiva	4	0 (0.0)	
Tobacco use			
No	44	6 (13.6)	0.432
Yes	60	5 (8.3)	
PORT			
No	52	4 (7.7)	0.336
Yes	52	7 (13.5)	
HPE Diagnosis			
WDSCC	73	4 (5.5)	0.008
MDSCC	31	7 (22.6)	
Margin status			
Clear	36	2 (5.6)	0.234
Close	68	9 (13.2)	
Lymphovascular Spread			
Absent	103	11 (10.7)	0.762
Present	1	0 (0.0)	
Perineural Invasion			
Absent	100	10 (10.0)	0.264
Present	4	1 (25.0)	
T Stage			
T1	54	2 (3.7)	0.017
T2	50	9 (18.0)	

^*δ*^Based on log-rank test.

**Table 3:** Cox regression analysis for risk of mortality in the studied patients.

Factors	Total sample *N*	Death n (%)	Hazard ratio (95% CI)	P value
Tobacco use				
No	44	6 (13.6)	1.00	0.842
Yes	60	5 (8.3)	0.87 (0.22, 3.51)	
HPE diagnosis				
WDSCC	73	4 (5.5)	1.00	
MDSCC	31	7 (22.6)	4.89 (1.19, 20.13)	0.028
Margin status				
Clear	36	2 (5.6)	1.00	
Close	68	9 (13.2)	1.17 (0.23, 5.88)	0.851
Perineural invasion				
Absent	100	10 (10.0)	1.00	
Present	4	1 (25.0)	1.71 (0.19, 15.20)	0.632
T stage				
T1	54	2 (3.7)	1.00	
T2	50	9 (18.0)	6.20 (1.27, 30.21)	0.024

**Table 4:** Univariate analysis for risk of recurrence in the population.

Factors	Total sample *N*	Locoregional recurrence *n* (%)	*P* value^*δ*^
Tobacco use			
Yes	60	5 (8.3)	0.740
No	44	5 (11.4)	
HPE diagnosis			
WDSCC	73	4 (5.5)	0.062
MDSCC	31	6 (19.4)	
Margin status			
Clear	36	2 (5.6)	0.488
Close	68	8 (11.8)	
Perineural invasion			
Absent	100	9 (9.0)	0.337
Present	4	1 (25.0)	
T stage			
T1	54	2 (3.7)	0.046
T2	50	8 (16.0)	

^*δ*^Using either chi-square test or Fisher's exact test as appropriate.

**Table 5:** Multivariate logistic regression model in predicting the recurrence in the sample with close and clear margin status.

Factors	Total sample *N*	Locoregional recurrence n (%)	Adjusted OR (95% CI)	P value
Tobacco use				
Yes	60	5 (8.3)	0.88 (0.18, 4.28)	0.876
No	44	5 (11.4)	1.00	
HPE diagnosis				
WDSCC	73	4 (5.5)	1.00	
MDSCC	31	6 (19.4)	4.21 (0.89, 19.93)	0.070
Margin status				
Clear	36	2 (5.6)	1.00	
Close	68	8 (11.8)	1.24 (0.22, 6.91)	0.810
Perineural invasion				
Absent	100	9 (9.0)	1.00	
Present	4	1 (25.0)	1.76 (0.10, 31.02)	0.696
T stage				
T1	54	2 (3.7)	1.00	
T2	50	8 (16.0)	5.41 (0.99, 29.36)	0.050

OR: odds ratio (95% CI: 95% confidence interval).
